# Predicting Low Cognitive Ability at Age 5—Feature Selection Using Machine Learning Methods and Birth Cohort Data

**DOI:** 10.3389/ijph.2022.1605047

**Published:** 2022-11-10

**Authors:** Andrea K. Bowe, Gordon Lightbody, Anthony Staines, Mairead E. Kiely, Fergus P. McCarthy, Deirdre M. Murray

**Affiliations:** ^1^ INFANT Research Centre, Cork, Ireland; ^2^ Department of Electrical and Electronic Engineering, University College Cork, Cork, Ireland; ^3^ School of Nursing, Psychotherapy, and Community Health, Dublin City University, Dublin, Ireland; ^4^ Cork Centre for Vitamin D and Nutrition Research, School of Food and Nutritional Sciences, University College Cork, Cork, Ireland; ^5^ Department of Obstetrics and Gynaecology, Cork University Maternity Hospital, Cork, Ireland; ^6^ Department of Paediatrics, Cork University Hospital, Cork, Ireland

**Keywords:** machine learning, cognition, prediction model, birth cohort, random forest

## Abstract

**Objectives:** In this study, we applied the random forest (RF) algorithm to birth-cohort data to train a model to predict low cognitive ability at 5 years of age and to identify the important predictive features.

**Methods:** Data was from 1,070 participants in the Irish population-based BASELINE cohort. A RF model was trained to predict an intelligence quotient (IQ) score ≤90 at age 5 years using maternal, infant, and sociodemographic features. Feature importance was examined and internal validation performed using 10-fold cross validation repeated 5 times. Results The five most important predictive features were the total years of maternal schooling, infant Apgar score at 1 min, socioeconomic index, maternal BMI, and alcohol consumption in the first trimester. On internal validation a parsimonious RF model based on 11 features showed excellent predictive ability, correctly classifying 95% of participants. This provides a foundation suitable for external validation in an unseen cohort.

**Conclusion:** Machine learning approaches to large existing datasets can provide accurate feature selection to improve risk prediction. Further validation of this model is required in cohorts representative of the general population.

## Introduction

Cognitive function is a broad construct consisting of multiple domains including learning, understanding, reasoning, problem solving, memory, language, attention, and decision making [1]. Early life is a crucial period for shaping the developing brain, and represents a window of both vulnerability and opportunity [2]. A failure to achieve early foundational cognitive skills may result in a permanent loss of opportunity to achieve full cognitive potential. This has significant adverse implications for outcomes throughout the life course including educational attainment [3], mental health [4], social mobility [5], financial well-being [6], and physical health [7].

Unlike children with more severe neurodevelopmental disorders who may be identified in infancy through routine developmental screening programmes, children with low cognitive ability may not display such overt deficits in early infancy [8]. Risk prediction models for poor cognitive outcomes in childhood have been widely studied among certain high risk populations such as pre-term and low birthweight infants [9]. However, far less has been published on their potential use among a general paediatric population.

Adverse cognitive outcomes in childhood are complex, heterogenous, and result from highly interactive relationships between biological, environmental, and social factors. The causative mechanisms are often poorly understood. This real-world complexity may be difficult to model with traditional statistical methods that rely on strong assumptions, which are often unrealistic in complex real world data [10]. Traditional regression methods can handle only a small number of predictors and require interaction terms to be specified *a priori* by the investigator, a process not commonly performed despite a wealth of evidence demonstrating the importance and strength of interactions between many of the relevant predictors, for example between prematurity and maternal education [11–13].

In contrast, machine learning (ML) aims to make a repeatable prediction by learning from patterns within the data, without prior assumptions or rules governing the process [14]. ML methods have the ability to handle many potential variables and to statistically model highly complex, non-linear, interactive relationships, without the need for prior manual specification of the interactions. With recent advances in both the quantity and quality of population based data available through large birth cohorts and electronic health records, the application of artificial intelligence and machine learning may assist in finding the optimal predictive patterns to enable early interventions [15]. To date, the application of ML for predicting childhood cognitive outcomes at a population level has not been explored.

Decision trees and random forests are one type of supervised ML algorithm. A decision tree resembles a flow chart whereby data is successively divided according to decisions based on the predictor variables (features) [16]. A random forest (RF) consists of multiple decision trees whose results are aggregated into a single result [16, 17]. One advantage of the RF is the relative “interpretability” when compared with other ML methods [18]. This means that it is possible to determine the relative importance of the features contained within the model and to explicitly interpret and describe important interactions. For many clinical applications the interpretability of the model for both clinicians and the public is important for successful implementation.

In this study we focus on the early identification of infants at risk of poor cognitive outcomes at 5 years of age in a general paediatric population. Acknowledging that interventions are more successful when implemented at the earliest possible stage, we focus on features which do not require invasive testing, and are readily available in the perinatal period at a population level. The objectives of this study are to determine the most important of these features for predicting low cognitive ability at age 5; to examine the important interactions between these features; to train a RF classification model using these features; and to examine the accuracy of this model within our cohort.

## Methods

### Study Population

**FIGURE 1:**
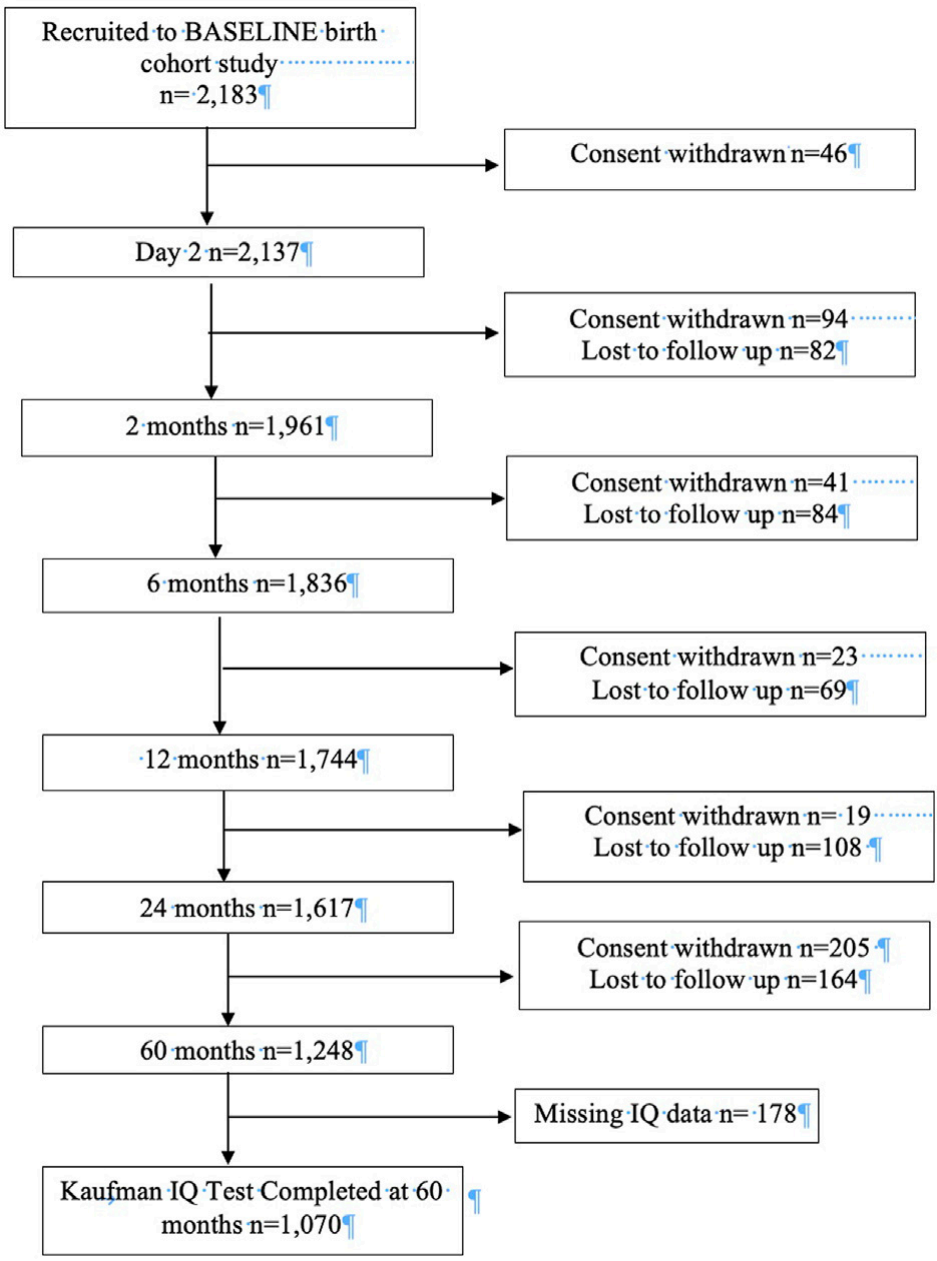
Flowchart describing BASELINE cohort and study population (Cork, Ireland. 2022). Legend: The flowchart details participation and attrition at each phase of the BASELINE birth cohort and describes how the study population for this study was arrived at.

The study population was mothers and infants from the Irish Cork BASELINE Study, the first longitudinal birth cohort in Ireland [19]. The BASELINE birth cohort was established to examine the effect of the *in-utero* and early life environment on health and neurodevelopmental outcomes in children [19]. Recruitment for BASELINE occurred through two streams. Stream 1 recruitment (*n* = 1,583) was from the Screening for Pregnancy Endpoints (SCOPE) pregnancy cohort, a multi-centre prospective study of low-risk, primiparous women which began in Cork in 2008, with the aim of examining adverse outcomes in pregnancy. All women who participated in the SCOPE study were invited to participate in the BASELINE birth cohort. Stream 2 recruitment (*n* = 600) began in 2010 in the postnatal wards of Cork University Maternity Hospital. Women who had a singleton pregnancy were invited to participate in BASELINE. Postnatal assessments were completed at day 2, and then at 2, 6, 12, 24 and 60 months. Infants with complete cognitive outcome data at age 5 years were eligible for inclusion in this study. A flowchart describing the BASELINE cohort and the participants included in this study is contained in Figure 1.

### Data Preprocessing

To create the training dataset, the data were preprocessed and transformed to a suitable format for model training. The main issues with the dataset were the imbalance and missing values.

The dataset was highly imbalanced with regard to the outcome of interest, meaning that the outcome classes of interest were not equally represented—specifically only a minority of the population fell into the outcome group of primary interest (low cognitive ability defined as an IQ ≤ 90). Learning from class-imbalanced data tends to produce a model bias in favour of the majority class (average/above cognitive ability), with poor predictive performance for the minority class (low cognitive ability) [20]. There are many different techniques to address the issue of class imbalance including over-sampling the minority class, under-sampling the majority class, boosting, bagging, and repeated random sub-sampling. Synthetic Minority Oversampling Technique (SMOTE) was used in this study, and involves a combination of over-sampling the minority class and under-sampling the majority class [21]. Unlike other oversampling techniques which simply duplicate minority class cases, SMOTE utilises an over-sampling approach where the minority class is over-sampled by creating synthetic examples [21]. This is achieved by taking each minority class sample and introducing random synthetic examples along the line segments that join it to a randomly selected sample from its k nearest minority class neighbours. This random convex combination of feature vectors is repeated until the desired amount of over-sampling, set by the user, is achieved [20, 21]. Under-sampling is achieved by randomly removing samples from the majority class. Through this combination the learning bias toward the majority class can be compensated for [20, 21]. The use of SMOTE is not without disadvantages—under-sampling may disregard potentially useful data points from the majority class in its attempt to rebalance the classes; the random selection of synthetic minority class data points used in over-sampling may produce distribution marginalisation [22].

Due to the different times of recruitment (antenatal recruitment vs. post-delivery recruitment), there were participants with missing data on features of interest measured in the antenatal period. As these data absences could not be determined to be at random, complete case analysis was performed. Imputation was also performed for missing predictor data using the standard random forest imputation method (missForest package) and analysis of imputed data is contained in online only material [23].

### Data Analysis

#### Outcome

The outcome of interest in this study was cognitive ability at 5 years of age. This was measured using the Kaufman Brief Intelligence Test Second Edition (KBIT-2), which was administered by trained research nurses at the 60-month follow up. It comprises three subtests: verbal knowledge and riddles, which measure verbal intelligence, and matrices, which measure non-verbal intelligence. The verbal knowledge subtest requires the child to point to a picture, from a selection of six, that best represents a vocabulary word spoken by the examiner. For the riddles subtest the child is asked to point to a picture or provide a word that solves a riddle spoken by the examiner. Together, these two subtests primarily measure crystalised verbal ability which comes from prior learning and experience [24]. The matrices subtest, which requires the child to choose a picture which follows a pattern or concept in pictures shown by the examiner, measures fluid reasoning and visual processing and is not dependent on knowledge of vocabulary or language [24].

The verbal and non-verbal scores are combined to provide a composite intelligent quotient (IQ). The Kaufmann test has been shown to be a reliable and valid measure of IQ in children and adults aged 4–90 years [24]. Composite IQ scores ≤90 were categorised “low cognitive ability” and scores above this as “average and above.” A cut-off of 90 corresponds to a cut-off of 1.5 standard deviations below the cohort mean IQ.

#### Features

A machine learning algorithm was trained to learn how to make this classification between low and average/above cognitive ability using the predictive features available in the dataset. The only inclusion criterion for predictive features was that the feature was easily measurable at a population level in the perinatal period. In total, there were 21 predictive features included in the study dataset. These were categorised into sociodemographic, lifestyle/behavioural, and birth/delivery and a description of measurement is provided (Supplementary Table S1). A correlation matrix (Supplementary Figure S1) was used to examine the correlation between features of interest and assess redundancy. All correlation coefficients were ≤0.70 and no features were deemed redundant.

The machine learning algorithm used for this classification problem was the Random Forest algorithm (using the “caret” and “randomForest” packages in R) [25, 26]. RF is an ensemble ML algorithm that is widely used in classification problems. A RF consists of multiple decision trees, each of which consists of a root node, split node(s), and terminal node(s). At each node a splitting step occurs whereby a decision is taken on how to partition the data for the nodes below, with each partition of data representing a branch in the tree [27]. At each node of the tree, a random subset of a defined number of features (mtry) is selected and only these variables are considered for partitioning the data at that node [11]. Splitting at nodes will continue down each branch until a stopping rule is satisfied, with this node being referred to as the leaf or terminal node [27]. To classify a participant, each tree will determine which terminal node that participant belongs to, culminating in a vote for that particular class. The forest predicts the class that gets the majority vote across all decision trees in the forest [26]. The RF algorithm allows for tuning of hyperparameters which control the learning process in the RF and these are discussed in Supplementary Text.

Feature importance, a measure of the degree of association between the feature and the classification result, was assessed using the Gini importance and the permutation accuracy importance. Gini impurity is a measure of how often an observation is incorrectly labelled if that labelling was performed at random [28]. A lower Gini impurity indicates a lower probability of misclassification. The Gini importance for a given node is the mean decrease in node impurity, weighted by the proportion of observations reaching that node in each decision tree in the forest [28]. A split which results in a large decrease of Gini impurity is important and the feature used for splitting will therefore have a high mean decrease in node impurity and a high Gini importance.

Bootstrap sampling of the training set for each tree means that each tree in the forest has its own “out of bag” (OOB) data which was not used in the construction of that particular tree [17]. The prediction accuracy is first measured on the OOB data. The values of the feature of interest are randomly shuffled and prediction accuracy is calculated again. The mean difference between the OOB errors before and after the random permutation of the feature is calculated. If a feature is important it would be expected that randomly changing its value would result in a stronger decrease in accuracy than the permutation of an unimportant variable [11, 29]. An alternative measure is the minimal depth of the feature which is described in Supplementary Material. The relationship between each feature and the outcome was examined using partial dependence plots, which provide a graphical representation of the marginal effect of a feature on the class probability.

The method used to evaluate the RF was repeated k-fold cross-validation. Performing k-fold cross validation involves first specifying a value for k, and in this study we chose to set it to k = 10, a value commonly used and supported by previous literature [30]. The dataset is shuffled randomly and split into 10 mutually exclusive folds. Each fold will function as a test set one time and will be used as part of the training set k-1 (or 9) times. This procedure is repeated a specified number of times, in this study 5 times, and the mean result across all folds and all repeats is reported [31]. A 10-fold cross validation repeated 5 times means that the accuracy estimates are performed across 50 different held-out test sets. The measures used in the evaluation were overall accuracy (proportion of correct predictions), sensitivity (proportion of those with low cognitive ability correctly identified), and specificity (proportion of those without low cognitive ability correctly identified), Recursive feature elimination (RFE) with 10-fold cross validation repeated 5 times was then used to train a parsimonious RF model (Model B) [32]. The performance was compared to the original RF model (Model A). Two logistic regression models, one containing all 21 predictors (Model C) and the other containing the 11 most important features (Model D) were also evaluated.

### Ethical Approval

The Clinical Research Ethics Committee of Cork Teaching Hospitals provided ethical approval for the BASELINE study (ref ECM5(9) 01/07/2008). It is registered at the United States National Institutes of Health Clinical Trials Registry (http://www.clinicaltrials.gov), ID: NCT01498965, and was carried out in line with the Declaration of Helsinki. The STROBE guidelines were followed in the conduct and reporting of this study [33].

## Results

### Demographics

There were 1,070 children who completed the KBIT-2 at a mean age of 60.8 months (standard deviation (SD) 1.7 months), and who were eligible for inclusion in this study. Composite IQ values ranged from 76–143, and a histogram is contained in Supplementary Figure S2. The characteristics of those who did and did not complete the KBIT-2 at 60 months are compared in Supplementary Table S2. There were 66 participants in the low cognitive ability group which had a mean IQ of 87.0, and 1,004 participants in the average/above group which had a mean IQ of 105.5.

The characteristics of participants according to cognitive ability group are described in Table 1. The association between each feature and the outcome is described using unadjusted odds ratios. Male participants had a mean IQ of 103.3, compared with females who had a mean IQ of 105.5. In the low cognitive ability group there was a significantly higher proportion of males compared with females (males 65.2% v females 34.8%, *p* = 0.022). For males, the odds of experiencing low cognitive ability at age 5 were 1.9 times higher compared with females (unadjusted odds ratio 1.88, 95% confidence interval 1.13–3.22). Other features which were associated with a significant increase in the odds of a child experiencing low cognitive ability were lower socioeconomic index, being in a lower income category, being from a single parent family, being formula fed, smoking in the first trimester of pregnancy, and living in government housing.

**TABLE 1:** Characteristics of those with low cognitive ability and those with average/above cognitive ability at 5 years of age (Cork, Ireland. 2022).

	Valid	Low cognitive ability^a^ (*n* = 66)	Average/above cognitive ability^a^ (*n* = 1,004)	*p*-value	Unadjusted odds of low cognitive ability OR (95%CI)
Sociodemographic					
Gender					
Female	1,070	23 (34.8)	504 (50.2)	0.022^b^	Ref
Male		43 (65.2)	500 (49.8)	0.022	1.88 (1.13–3.22)
Maternal Relationship Status	1,070				
In a relationship		60 (90.9)	973 (96.9)		Ref
Not in a relationship		6 (9.1)	31 (3.1)	0.025^b^	3.14 (1.15–7.32)
Maternal migration hx	1,059				
Lived in country >2 generations		55 (84.6)	870 (87.5)		Ref
Mother immigrated		7 (10.8)	94 (9.5)		1.18 (0.48–2.50)
One or both parents immigrated		3 (4.6)	30 (3.0)	0.534^c^	1.58 (0.37–4.62)
Ethnicity					
European	1,070	65 (98.4)	993 (98.9)		Ref
Other		1 (1.6)	11 (1.1)	0.536^c^	0.08–7.31
Total years of schooling	1,070	13.2 (0.8)	13.4 (0.8)	0.109^d^	0.75 (0.56–1.03)
Maternal Employment Status	1,067				
Full time		49 (75.4)	822 (82.0)		Ref
Part time		9 (13.8)	89 (8.9)		1.70 (0.76–3.41)
Student		1 (1.5)	15 (1.5)		1.12 (0.06–5.69)
Homemaker		0 (0.0)	37 (3.7)		0.00 (0.00–3.15)
Unemployed/sickness benefit		6 (9.2)	39 (3.9)	0.061^c^	2.58 (0.95–5.98)
Socioeconomic index—mean (sd)	1,070	37.3 (15.1)	44.5 (15.5)	<0.001^d^	0.97 (0.95–0.98)
Accommodation type	1,070				
Own house/flat		46 (69.7)	797 (79.4)		Ref
Private rental		14 (21.2)	172 (17.1)		1.41 (0.73–2.56)
Government/council rental		4 (6.1)	13 (1.3)		5.33 (1.46–15.76)
Other		2 (3.0)	22 (2.2)	0.026^a^	1.58 (0.25–5.57)
Family Income	1,042				
<21 k		8 (12.7)	45 (4.6)		Ref
21–42 k		14 (22.2)	164 (16.7)		0.48 (0.19–1.27)
43–63 k		16 (25.4)	217 (22.1)		0.41 (0.17–1.08)
64–84 k		16 (25.4)	249 (25.4)		0.36 (0.15–0.94)
85–105		5 (7.9)	158 (16.1)		0.18 (0.05–0.56)
106–140 k		4 (6.3)	122 (12.4)		0.18 (0.05–0.62)
>140 k		0 (0.0)	25 (2.6)	0.042^c^	0.0 (0.0–0.0)
Maternal age (years)—mean (sd)	806	29.7 (4.3)	30.7 (3.9)	0.123^d^	0.93 (0.86–1.01)
Behavioural/Lifestyle					
Maternal BMI	806	26.1 (4.6)	24.9 (4.0)	0.115^d^	1.07 (0.99–1.14)
Depression score^f^– median (IQR)	795	6.0 (7.5)	5.0 (7.0)	0.743^e^	1.02 (0.95–1.09)
Units of alcohol/week 1st trimester—median (IQR)	806	4.0 (8.4)	2.9 (5.1)	0.127^e^	1.07 (1.02–1.11)
Cigarettes/day in 1st trimester—median (IQR)		0 (5.0)	0 (0.0)	0.042^e^	1.08 (1.02–1.13)
Birth/Delivery					
Apgar score at 1 min—median (IQR)	1,061	9.0 (0.0)	9.0 (0.0)	0.773^e^	0.94 (0.79–1.14)
Apgar score at 5 min—median (IQR)	1,061	10.0 (1.0)	10 (1.0)	0.877^e^	1.08 (0.78–1.61)
Birth head circumference (cm)—mean (sd)	1,055	35.1 (1.4)	34.9 (1.5)	0.287^d^	1.09 (0.92–1.30)
Admission to NICU	1,070				
Not Admitted		62 (93.9)	917 (913)		Ref
Admitted		4 (6.1)	87 (8.7)	0.648^b^	0.68 (0.20–1.70)
Infant gestational age	1,070	39.5 (1.75)	40.0 (2.0)	0.155^e^	0.90 (0.78–1.05)
Infant birthweight (g)—mean (sd)		3,546.2 (1.6)	3,526.8 (1.5)	0.183^d^	1.00 (0.99–1.00)
Infant feeding on discharge	1,061				
Predominantly breastfed		21 (31.8)	460 (46.2)		Ref
Partially breastfed		19 (28.8)	289 (29.0)		1.44 (0.76–2.73)
Formula fed		26 (39.4)	246 (24.7)	0.018^b^	2.32 (1.28–4.24)
High Risk Birth	833	1 (2.2)	67 (8.5)	0.167	0.24 (0.01–1.15)
Cognitive					
IQ—mean (sd)	1,070	87.0 (3.4)	105.5 (7.8)	<0.001	

OR, odds ratio; CI, confidence interval.

^a^ Data described as n (%) except where otherwise indicated.

^b^ *p*-value calculated using Pearson’s Chi-square test.

^c^ *p*-value calculated using Fishers’ Exact test.

^d^ *p*-value calculated using Welch Two Sample *t*-test.

^e^ *p*-value calculated using Wilcoxon rank sum test.

^f^ Measured at 15 weeks gestation using Edinburgh Depression Scale.

### Machine Learning

After applying complete case analysis there were 36 participants (5.0%) in the low cognitive ability group and 683 (95.0%) in the average/above group. SMOTE was applied with 600% over-sampling to the minority class and 200% under-sampling to the majority class. The rebalanced dataset contained 252 participants (36.8%) and 432 (63.2%) in the low and average/above groups, respectively.

**FIGURE 2:**
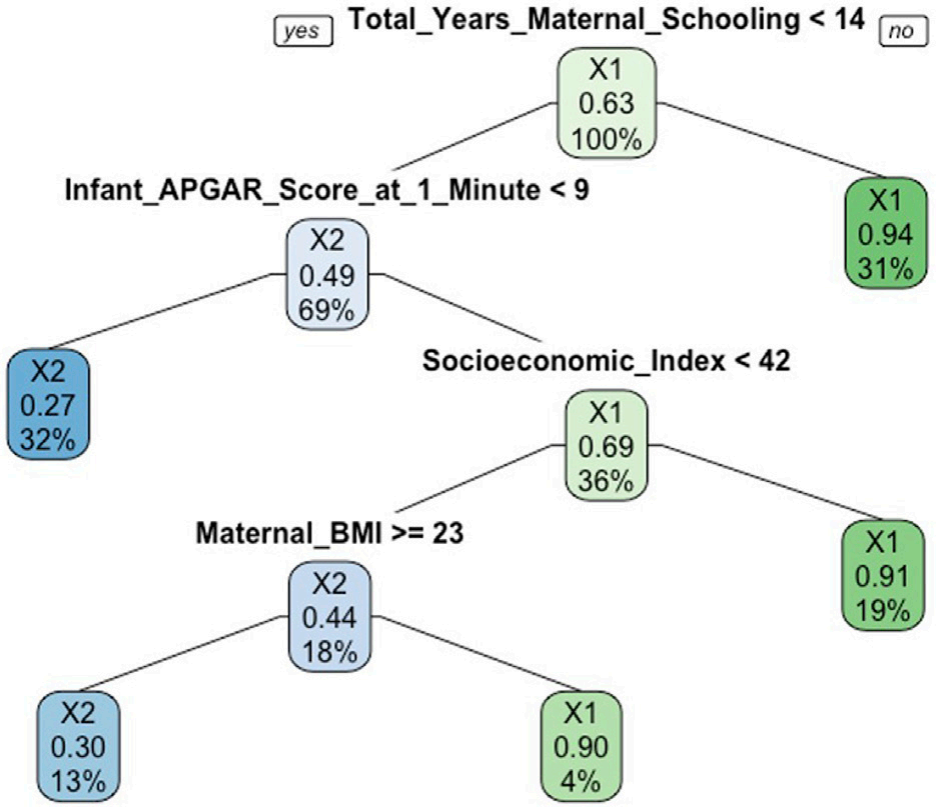
Example decision tree from the random forest model predicting low cognitive ability at age 5 (Cork, Ireland. 2022). Legend: X1 refers to the “average/above cognitive ability” group and X2 refers to the “low cognitive ability” group. At the top of decision tree, the overall probability of “average/above” cognitive ability is shown. 63% of participants had average/above cognitive ability (after SMOTE applied). The root node asks whether the total years of maternal schooling was <14. If no, then you proceed down the right branch. 31% had ≥14 years of schooling and the probability of average/above cognitive ability was 94%. If yes, then proceed down left branch. 69% had <14 years and a probability of average/above cognitive ability of 49%. The next splitting node asks whether the Apgar score at 1 min was <9. If yes, then proceed down left branch to the leaf or terminal node. 32% had an Apgar score <9 at 1 min and a probability of average/above cognitive ability of 27%. If no, then proceed down the right branch where the next splitting node asks whether the socioeconomic index was less than 42.

The results of the final RF model containing 21 features (Model A) are presented in Table 2, alongside the parsimonious model containing 11 features (Model B) and logistic regression models (Models C and D). Of note, the 11 features identified on RFE for the parsimonious model were contained within the 13 most important features consistently identified using the random forest importance measures. The accuracy of Model B was 0.95 and did not improve by more than 0.01 following inclusion of further features beyond these 11. The parsimonious Model B achieved a sensitivity of 0.89 and a specificity of 0.98 on 10-fold cross validation To illustrate how a RF is constructed, an example decision tree from the Model A is shown in Figure 2.

**TABLE 2:** Results of evaluation of models A-D (Cork, Ireland. 2022).

	Method	Features	Mtry	Accuracy	Sensitivity	Specificity
Model A	Random forest	21	4	0.95	0.89	0.99
Model B	Random forest	11	5	0.95	0.89	0.98
Model C	Logistic regression	21	NA	0.78	0.64	0.86
Model D	Logistic regression	11	NA	0.77	0.48	0.83

Model A: Random forest model using 21 features (total years schooling, Apgar score 1 min, socioeconomic index, Apgar score 5 min, family income, gestational age, units of alcohol in first trimester, head circumference at birth, maternal age, maternal depression score, accommodation type, infant gender, maternal relationship status, migration history, maternal employment status, admission to NICU, accommodation type, infant feeding on discharge, high risk birth).

Model B: Parsimonious random forest model using 11 features (total years schooling, Apgar score 1 min, socioeconomic index, maternal BMI, Apgar score 5 min, family income, gestational age, units of alcohol in first trimester, head circumference at birth, maternal age, maternal depression score).

Model C: Logistic regression model using 21 features.

Model D: Logistic regression model using 11 features.

**FIGURE 3:**
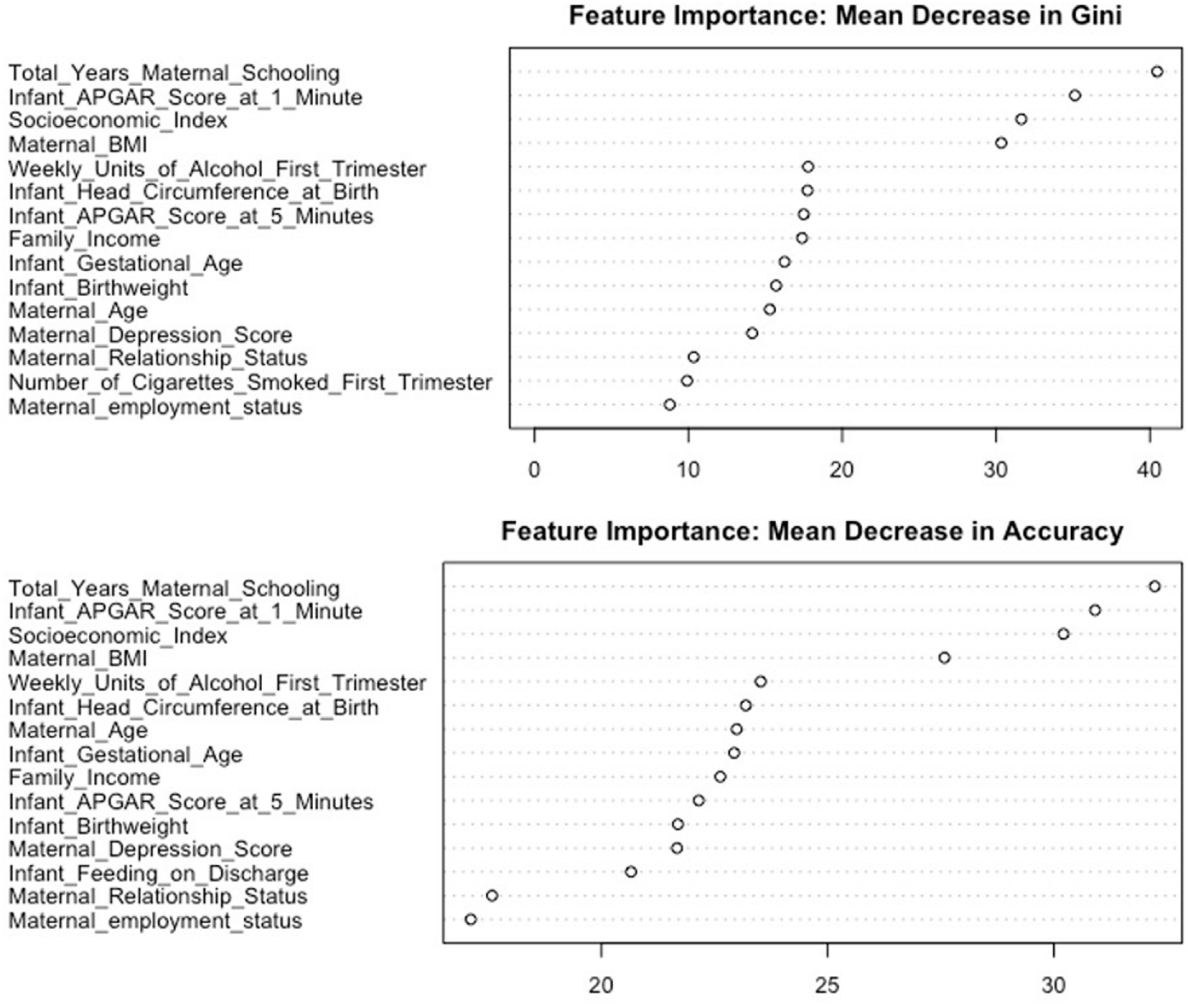
Feature importance plot showing the mean decrease in Gini and mean decrease in accuracy for the 15 most important features (Cork, Ireland. 2022). Legend: The top plot in the figure shows the mean decrease in Gini for 15 features ranked in descending order of importance. A feature which results in a larger decrease in Gini impurity is a more important feature. The bottom plot in the figure shows the mean decrease in accuracy for 15 features ranked in descending order of importance. A feature with a larger mean decrease in accuracy is a more important feature.

There were 13 features identified consistently across measures of feature importance (Figure 3 and Supplementary Figure S3). These were total years maternal schooling, socioeconomic index, family income, maternal age, maternal relationship status, maternal BMI, weekly units of alcohol in first trimester, maternal depression score, Apgar score at 1 min, Apgar score at 5 min, infant birthweight, infant gestational age, and infant head circumference. The correlations between importance rankings for the 21 features are described in Supplementary Figure S4. The relationship between each of these features and the probability of low cognitive ability can be visualised in the partial dependence plots in Supplementary Figure S5.

Using these variables a dataframe of the most important interactions in the model, determined by occurrence was generated. Table 3 outlines the six interaction terms which occurred most frequently in Model A. The mean minimal depth refers to the mean minimal depth of the interacting feature conditional on the main feature being the root node. The unconditional mean minimal depth is the mean minimal depth of the feature without stipulating this root. An interaction term with a lower mean minimal depth indicates the interaction is a more important feature than either feature alone [34]. For example, the interaction between total years of maternal schooling and weekly units of alcohol in the first trimester was a more important predictive feature than weekly units of alcohol or total years of maternal schooling alone.

**TABLE 3:** Six most frequently occurring interactions in random forest model model A (Cork, Ireland. 2022).

Main feature	Interacting feature	Occurrences	Unconditional minimal depth of interacting feature	Mean minimal depth of interacting feature conditional on main feature
Total Years Schooling	Weekly units alcohol first trimester	334	3.21	2.0
Total Years Schooling	Maternal BMI	333	2.71	2.14
Total Years Schooling	Infant Birthweight	329	3.73	2.43
Total Years Schooling	Maternal Depression Score	325	3.78	2.73
Apgar Score 1 Minute	Weekly units alcohol in first trimester	324	3.21	2.18
Total Years Schooling	Infant head circumference	321	3.68	2.72

## Discussion

In this study we identified the most important features for predicting low cognitive ability at age 5 from a range of features which could be easily measured in the perinatal period at a population level. There were 13 features consistently identified across the range of importance measures used - total years maternal schooling, socioeconomic index, family income, maternal age, maternal relationship status, maternal BMI, weekly units of alcohol in first trimester, maternal depression score, Apgar score at 1 min, Apgar score at 5 min, infant birthweight, infant gestational age, and infant head circumference.

No previous studies using ML methods for prediction of cognitive outcomes in childhood among a general paediatric population have been identified in the literature for comparison. However, the multivariable prediction models that have been developed for similar outcomes using traditional linear and logistic regression methods identified many of the same predictors including maternal education, socioeconomic status, income, and employment status [35–37]. An advantage of this study is that through use of feature importance metrics, partial dependence plots, and an exploration of the most important interactions, a model that is relatively interpretable was developed where one can understand the decision logic of the model. In addition, an explanation of the key components required to understand the development and interpretation of a ML model were provided.

The findings of the study are subject to limitations. Bias in ML models can arise in data collection, model development, and model evaluation [38]. First, the BASELINE cohort is subject to sampling bias as the majority of those recruited were low-risk, primiparous women who may not be representative of the pregnant population as a whole. In addition, those who remain engaged in cohort studies systematically differ from those who do not. As shown in Supplementary Table S2 participants whose child completed IQ assessments at age 5 were more likely to be older non-smokers, with higher education and lower depression scores, and were more likely to have infants with a higher birthweight, head circumference, and gestational age. The generalisability, equity, and utility of a predictive model at a population level is dependent on data that is representative of the population [39].

Sampling bias, such as that outlined here, is a significant challenge in the application of ML methods to address population health problems. Often the most vulnerable populations, including the homeless, intravenous drug users, and ethnic minorities, are absent from large cohort studies, but may be among the populations who would benefit most from optimised prediction models. As the potential to collect large quantities of data increases, through electronic health records, electronic devices, and wearable technology, researchers must ensure that minority groups are represented and barriers to participation which may be cultural, financial, linguistic, or time-bound must be removed.

The evaluation of a ML model should be performed across diverse patient groups, on data that was not used in the training process. In this study external validation on an unseen cohort was not performed and therefore the performance metrics of the model should be interpreted with caution. As the dataset used in this study was imbalanced with regard to the outcome of interest the entire dataset was used in the training process and internal validation only was applied. The next step is to now validate this model using an unseen cohort, ideally one that is more representative of the population as a whole.

A further limitation associated with the use of pre-existing birth cohort data is that features, such as genetic information and parental IQ, which have an important causal relationship with the outcome of interest and would likely improve predictive power, may not have been collected [40]. However, a model relying on genetic data would not be feasible to implement at a population level.

ML was used in this study to optimise the predictive power of the model. Risk prediction and early intervention is a form of secondary prevention, and should occur in conjunction with primary prevention strategies to reduce the causative factors. However, a limitation of ML is that it does not inform us of the causal relationship between the features and the outcome. While many of the predictive features identified in this study, such as parental education [41], have an existing literature base to demonstrate a causal relationship with the outcome, predictors should not be assumed to be causal factors.

Finally, there are important ethical issues to be considered. Identifying children at risk of poor cognitive outcomes risks labelling a child early in life. It is well documented that early labelling can negatively impact both parent and teacher expectations, as well as the child’s own self-concept [42, 43]. These risks are especially pertinent for children and families who may be incorrectly identified as at risk on a screening or risk prediction tool and who are unlikely to benefit from early intervention. For those correctly identified, the question is whether these risks are outweighed by the benefit of providing early intervention.

In conclusion, the application of machine learning to address population health challenges has received much less attention than its application in the clinical setting. Disparities in cognitive development can be seen as early as 2 years of age, but unabated will amplify over time, making early intervention essential [44]. The socioenvironmental exposures of a child in early life are modifiable, and enrichment of the early environment has been shown to be both feasible, achievable, and effective in improving cognitive outcomes, if appropriately resourced [45, 46]. However, the effectiveness of such intervention programmes is dependent upon the early and accurate identification of the children who are most likely to benefit.

Current population based strategies which rely on the presence of a delay in development prior to intervention miss the opportunity for pre-emptive intervention in the period of optimal neuroplasticity, and risk missing the most vulnerable who may not present to routine screening. The perinatal period is a unique window of opportunity where there is almost universal contact between the public and healthcare professionals, and is an ideal time for engagement with high-risk mother-infant dyads. As clinical disciplines strive toward a personalised approach to medicine, population health must not be left behind. Investigation into how machine learning can be used to assist in addressing disparities in cognitive development in early childhood—a significant population health challenge, rooted in the social determinants of health and exacerbated by inequity—requires further research. This targeted approach must be considered in the context of wider changes to educational policy, planning policy, and economic policy which have a central role to play in addressing disparities in childhood development.

## Data Availability

The BASELINE data are not currently publicly available due to ethical restrictions. Application for the use of the data in collaborative and ethically approved projects can be made through the INFANT centre website or by contacting infant@ucc.ie.
