# Supplementary material for: Predicting Low Cognitive Ability at Age 5—Feature Selection Using Machine Learning Methods and Birth Cohort Data
Source: Int J Public Health. 2022 Nov 10;67:1605047. doi: 10.3389/ijph.2022.1605047 (PMC9684182; doi:10.3389/ijph.2022.1605047)
Supplement: Supplementary file 1 [file DataSheet1.docx]

**Supplemental Files**

Table S1. Features considered for random forest model predicting low cognitive ability at age 5 (Cork, Ireland. 2022)

| **Category** | **Feature** | **Time of measurement** | **Description** | **Type** |
| --- | --- | --- | --- | --- |
| Sociodemographic | Maternal Age | 15 wks^*^ gestation | Age in years | Numeric |
|  | Maternal total years of schooling | 2 months^^^ | Total years of schooling for mother | Numeric |
|  | Socioeconomic index | 2 months | New Zealand Socioeconomic Index Guide | Numeric |
|  | Accommodation type | 2 months | own house/flat, private rental, council/government rental, other | Factor |
|  | Family income | 2 months | <21k, 21-42k, 43-63k, 64-84k, 85-105k, 106-140k, >140k | Factor |
|  | Maternal relationship status | 2 months | In a relationship, not in a relationship | Factor |
|  | Maternal employment status | 2 months | Full time, Part time, Student, Homemaker, Unemployed/sickness | Factor |
|  | Maternal migration history | 2 months | Mother immigrated, One or both parents immigrated, Lived in country >2gen | Factor |
| Behavioural |  |  |  |  |
|  | Maternal BMI | 15 wks gestation | Measured weight in kg/measured height in m^2^ | Numeric |
|  | Alcohol intake first trimester | 15 wks gestation | Units of alcohol per week in first trimester | Numeric |
|  | Cigarettes smoked in first trimester | 15 wks gestation | Number of cigarettes per day in first trimester | Numeric |
|  | Depression score | 15 wks gestation | Edinburgh Postnatal Depression Scale | Numeric |
| Birth/Delivery | Birthweight | Birth^+^ | Birthweight in grams | Numeric |
|  | Admission to Neonatal Intensive Care Unit | Birth | Yes, No | Factor |
|  | High risk birth | Birth | Yes, No | Factor |
|  | Gestational age | Birth | Gestational age in weeks | Numeric |
|  | Apgar score 1 minute | Birth | Apgar score measured at 1 minute | Numeric |
|  | Apgar score 5 minutes | Birth | Apgar score measured at 5 minutes | Numeric |
|  | Head circumference at birth | Birth | Head circumference in cms | Numeric |
|  | Infant feeding at discharge | Birth | Predominantly breastfed, Partially breastfed/mixed fed, Formula fed | Factor |
|  | Infant Gender | Birth | Male, Female | Factor |

^*^ 15 weeks gestation; ^^^ Infant age 2 months; ^+^ Day 2 after birth

Figure S1. Correlation matrix demonstrating correlation between features of interest (Cork, Ireland. 2022)


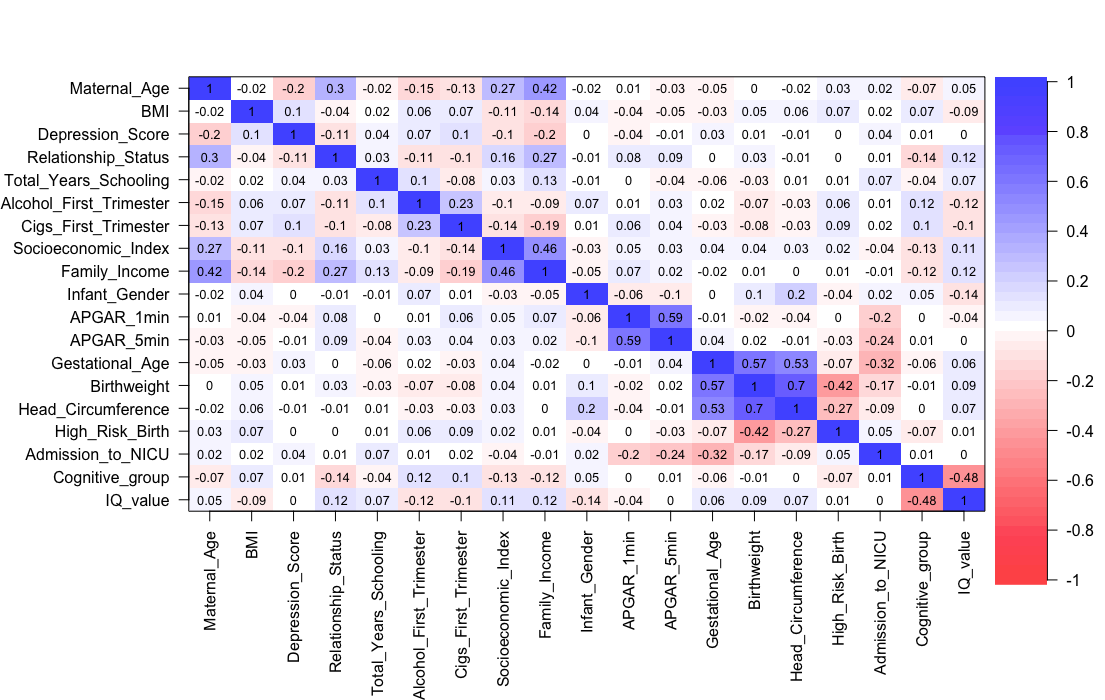


Figure S2. Histogram of composite IQ scores at age 5 in the BASELINE cohort (Cork, Ireland. 2022)


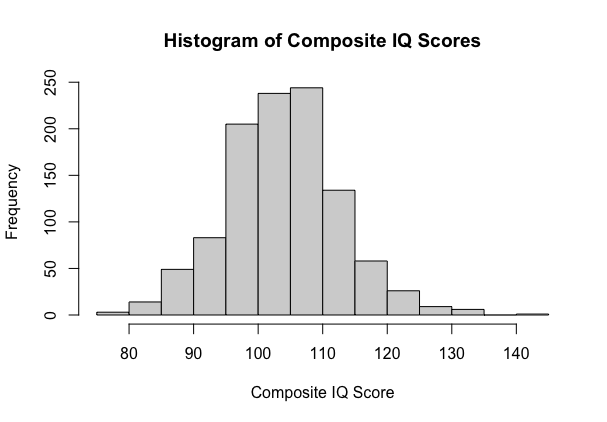


Table S2. Sociodemographic, behavioural/lifestyle and birth/delivery characteristics of those who did and did not complete KBIT-2 at 60 months (Cork, Ireland. 2022)

|  | Valid | Completed IQ test at 60 months  (n= 1,070) | Valid | Did not complete IQ test at 60 months  n = (1,113) |  |
| --- | --- | --- | --- | --- | --- |
| **Sociodemographic** |  |  |  |  |  |
| **Gender** |  |  |  |  |  |
| Male | 1,070 | 545 (50.9) | 1,113 | 555 (49.9) |  |
| Female |  | 525 (49.1) |  | 558 (50.1) | 0.648 |
| **Relationship Status** | 1,070 |  |  |  |  |
| In a relationship |  | 1,032 (96.4) |  | 816 (92.5) |  |
| Not in a relationship |  | 38 (3.6) |  | 66 (7.5) | <0.001 |
| **Maternal migration hx** | 1,059 |  | 871 |  |  |
| Mother immigrated |  | 102 (9.6) |  | 183 (21.0) |  |
| One parent immigrated |  | 15 (1.4) |  | 14 (1.6) |  |
| Both parents immigrated |  | 18 (1.7) |  | 21 (2.4) |  |
| Lived in country >2 generations |  | 924 (87.3) |  | 653 (75.0) | <0.001 |
| Total years of schooling | 1,070 | 13.41 (0.76) | 882 | 13.16 (0.97) | <0.001 |
| **University complete/attending** | 1,069 |  | 882 |  |  |
| Yes |  | 612 (57.2) |  | 447 (50.7) |  |
| No |  | 457 (42.8) |  | 435 (49.3) | 0.004 |
| **Current Job Status** | 1,067 |  | 880 |  |  |
| Full time |  | 869 (81.4) |  | 635 (72.2) |  |
| Part time |  | 99 (9.3) |  | 110 (12.5) |  |
| Student |  | 16 (1.5) |  | 23 (2.6) |  |
| Homemaker |  | 37 (3.5) |  | 53 (6.0) |  |
| Unemployed/sickness benefit |  | 46 (4.3) |  | 59 (6.7) | <0.001 |
| **Socioeconomic index – mean (sd)** | 1,070 | 44.13 (15.56) | 882 | 40.82 (16.34) | <0.001 |
| **Accommodation type** | 1,070 |  | 882 |  |  |
| Own house/flat |  | 843 (78.8) |  | 553 (62.8) |  |
| Private rental |  | 186 (17.4) |  | 267 (30.3) |  |
| Government/council rental |  | 17 (1.6) |  | 31 (3.5) |  |
| Other |  | 24 (2.2) |  | 30 (3.4) | <0.001 |
| **Family Income** | 1,042 |  | 833 |  |  |
| <21k |  | 53 (5.1) |  | 103 (12.4) |  |
| 21-42k |  | 178 (17.1) |  | 183 (22.0) |  |
| 43-63k |  | 233 (22.4) |  | 203 (24.4) |  |
| 64-84k |  | 263 (25.2) |  | 152 (18.2) |  |
| 85-105 |  | 164 (15.7) |  | 110 (13.2) |  |
| <106k |  | 151 (14.5) |  | 82 (9.8) | <0.001 |
| Maternal age (years) – mean (sd) | 806 | 30.7 (3.9) | 777 | 29.25 (4.82) | <0.001 |
| **Behavioural/Lifestyle** |  |  |  |  |  |
| Maternal BMI | 806 | 24.97 (4.06) | 777 | 24.80 (4.21) | 0.415 |
| Depression score at 15 weeks gestation – mean (sd) | 795 | 4.92 (4.26) | 768 | 5.50 (4.35) | 0.008 |
| Depression score at 15 weeks gestation – median (IQR) | 795 | 4 (7) | 768 | 5 (6) | 0.004 |
| Units of alcohol/week 1^st^ trimester – mean (sd) | 806 | 4.49 (5.40) | 777 | 5.07 (7.19) | 0.070 |
| Units of alcohol/week 1^st^ trimester – median (IQR) | 806 | 3.0 (5.0) | 777 | 3 (5.8) |  |
| **Smoking status pre pregnancy** | 806 |  | 777 |  |  |
| Non smoker |  | 612 (75.9) |  | 532 (68.5) |  |
| Smoker |  | 194 (24.1) |  | 245 (31.5) | 0.001 |
| **Smoking status 1^st^ trimester** | 806 |  |  |  |  |
| Non smoker |  | 628 (77.9) |  | 546 (70.3) |  |
| Smoker |  | 178 (22.1) |  | 231 (29.7) | <0.001 |
| **Birth/Delivery** |  |  |  |  |  |
| Apgar score at 1 minute – mean (sd) | 1,061 | 8.5 (1.2) | 1,099 | 8.5 (1.2) | 0.892 |
| Apgar score at 1 minute – median (IQR) | 1,061 | 9.0 (0.0) | 1,099 | 9 (0.0) | 0.873 |
| Birth head circumference (cm) – mean (sd) | 1,055 | 34.90 (1.51) | 1,099 | 34.75 (1.62) | 0.031 |
| **Admission to NICU** | 1,070 |  |  |  |  |
| Not Admitted |  | 996 (93.1) |  | 1,012 (90.9) |  |
| Admitted |  | 74 (6.9) |  | 101 (9.1) | 0.075 |

Figure S3. Feature importance plot showing the mean minimal depth of the 15 most important features (Cork, Ireland. 2022)


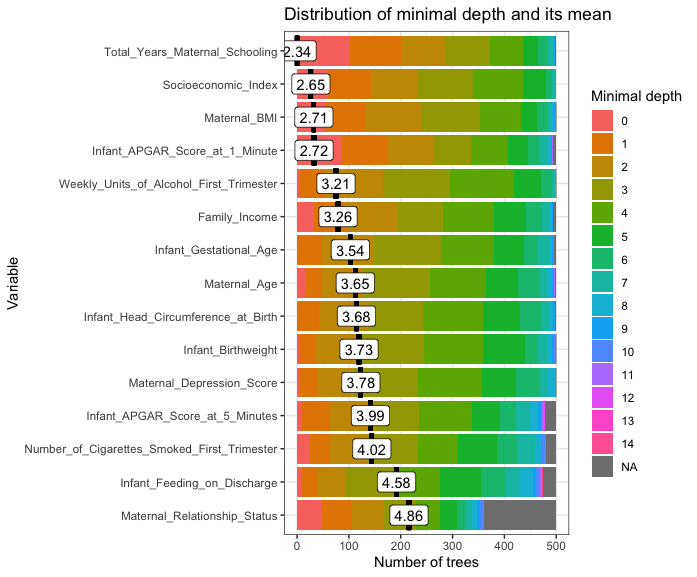


Figure S4. Plot of correlations between importance rankings for 21 features according to different measures of importance (Cork, Ireland. 2022)


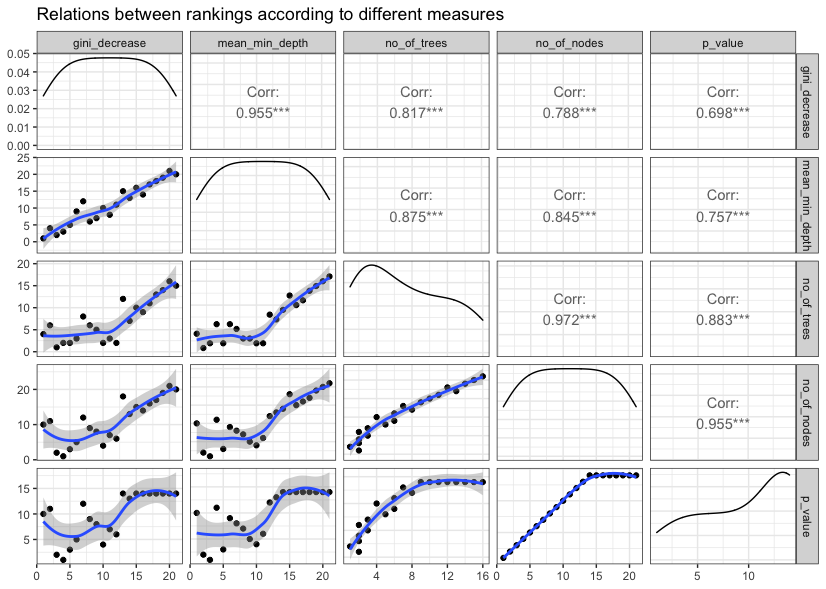


Figure S5. Partial dependence plots showing relationship between 11 important features and the probability of low cognitive ability (Cork, Ireland. 2022)


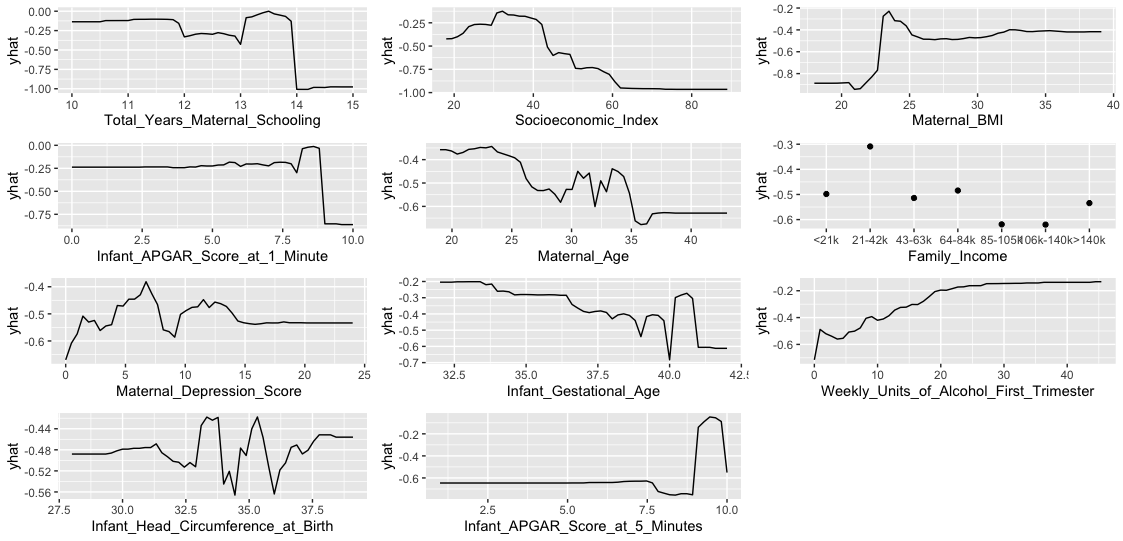


**Supplemental Data Analysis**

Analysis of Imputed Dataset

Methods

There were 1,070 participants in the BASELINE study who completed the Kaufman composite IQ test at age 5. Imputation was performed with the ’missForest’ package, which uses a random forest imputation algorithm. SMOTE was then applied to the imputed dataset to address the class imbalance.

Results

Shown below are the 15 most important features identified using mean decrease in Gini and accuracy based on this dataset. The random forest model was trained using the ‘caret package’ with 10-fold cross validation repeated 5 times. The optimal mtry was 4.

Based on 10-fold cross validation repeated 5 times, the accuracy of the model was 91.7%. The optimal model achieved a sensitivity of 86.1% and a specificity of 96.1%.

There were some differences in the fifteen most important features identified using complete case analysis and imputation and these are shown in Figure S6. The 13 features identified consistently using complete case analysis, apart from maternal relationship status, were all among the 15 most important variables identified using the imputed dataset.

Figure S6. Feature importance plot showing the mean decrease in Gini and mean decrease in accuracy for the 15 most important features based on imputed dataset (Cork, Ireland. 2022)


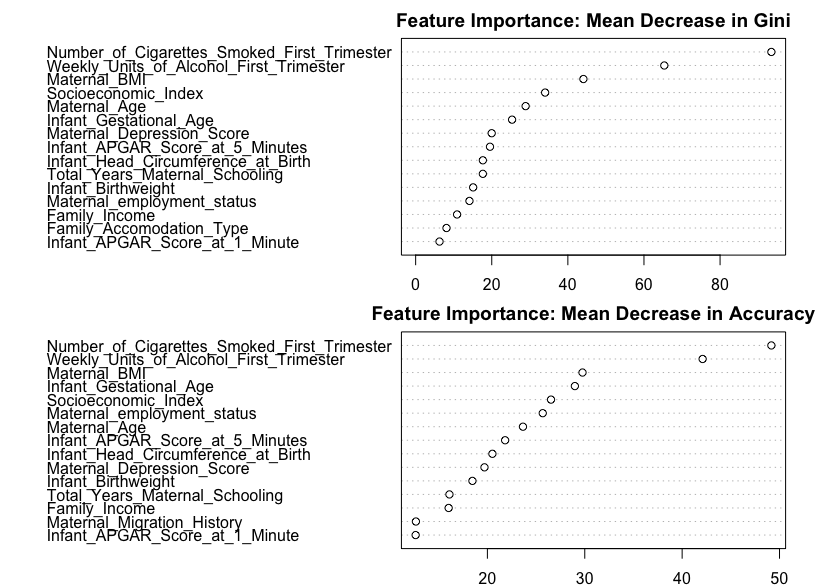


**Supplemental Text**

**Hyperparameters**

Hyperparameters refer to the parameters that can be “tuned” with the aim of improving model performance.^47^ Default parameters are set by statistical packages but can be altered through methods such as grid-searching. The parameter ‘mtry’ refers to the number of features selected at random as candidate features at each split.^11^ A low value can ensure heterogenous, less correlated trees, and may increase the selection of features with small or moderate effects that can contribute to predictive performance, but which otherwise may be masked by features with stronger effects.^11^ A large mtry may ensure features with stronger predictive ability are selected.^11^

The number of trees in a forest, ntree, is not a tuning parameter as such but should be set sufficiently high so that each candidate feature has opportunity to be selected.^48^ However it has been shown that there is likely an optimal threshold for the number of trees, beyond which there is no performance gain and only computational cost. The parameter ‘nodesize’, specifies the minimum number of observations at a terminal node. A lower value increases the number of splits, and therefore the depth of the tree, whereas a higher value results in a smaller tree. ^11^ Sample size refers to the number of observations used for the training of each tree and replace refers to whether the observations are replaced after each sample is randomly selected.^49,50^ With replacement, bootstrap samples are created whereas subsamples of the data are created if observations are not replaced. ^11^

**Feature Importance: Mean Minimal Depth**

The minimal depth refers to the depth of the node which splits on that feature. Features with high importance are those that tend to divide large numbers of observations and these nodes tend to be present in the initial levels of a tree. The mean minimal depth averages the depth of the splitting node for that feature across all trees in the forest and a lower value is likely to indicate a more important feature. ^49^

**Additional References**

41 Rindermann, H. & Ceci, S. J. Parents’ education is more important than their wealth in shaping their children’s intelligence: Results of 19 samples in seven countries at different developmental levels. *Journal for the Education of the Gifted* 2018; 41(4), 298–326.

42 Jussim L, Harber KD. Teacher expectations and self-fulfilling prophecies: knowns and unknowns,  resolved and unresolved controversies. *Personality and social psychology review : an official journal of the Society for  Personality and Social Psychology, Inc* 2005; **9**: 131–155.

43 Taylor LM, Hume IR, Welsh N. Labelling and self‐esteem: the impact of using specific vs. generic labels. *Educational Psychology* 2010; **30**: 191–202.

44 von Stumm S, Plomin R. Socioeconomic status and the growth of intelligence from infancy through adolescence. *Intelligence* 2015; **48**: 30–36.

45 Love JM, Chazan-Cohen R, Raikes H, Brooks-Gunn J. What makes a difference: Early Head Start evaluation findings in a developmental  context. *Monographs of the Society for Research in Child Development* 2013; **78**: vii–viii, 1–173.

46 Troller-Renfree S v, Costanzo MA, Duncan GJ, Magnuson K, Gennetian LA, Yoshikawa H *et al.* The impact of a poverty reduction intervention on infant brain activity. *Proceedings of the National Academy of Sciences* 2022; **119**: e2115649119.

47 Huang BFF, Boutros PC. The parameter sensitivity of random forests. *BMC bioinformatics* 2016; **17**: 331.

48 Oshiro TM, Perez PS, Baranauskas JA. How Many Trees in a Random Forest? BT  - Machine Learning and Data Mining in Pattern Recognition. In: Perner P (ed). . Springer Berlin Heidelberg: Berlin, Heidelberg, 2012, pp 154–168.

49 Probst P, Wright M, Boulesteix A-L. Hyperparameters and Tuning Strategies for Random Forest. *arXiv* 2019.

50 Aria M, Cuccurullo C, Gnasso A. A comparison among interpretative proposals for Random Forests. *Machine Learning with Applications* 2021; **6**: 100094.
